# Supplementary material for: Variation in Survival and Gut Microbiome Composition of Hatchery-Grown Native Oysters at Various Locations within the Puget Sound
Source: Microbiol Spectr. 2022 May 10;10(3):e01982-21. doi: 10.1128/spectrum.01982-21 (PMC9241838; doi:10.1128/spectrum.01982-21)
Supplement: SUPPLEMENTAL FILE 1 — Supplemental material. Download spectrum.01982-21-s001.pdf, PDF file, 0.3 MB [file spectrum.01982-21-s001.pdf]

Supplemental Table A. Statistical tests and results for Shannon's alpha diversity index. Table A1 demonstrates results from a Kruskal Wallis non-parametric one-way analysis of variance across sample types (seawater, sediment, shell biofilm, and oyster gut) and the pairwise comparisons for each sample type. Table A2 demonstrates results from a Kruskal Wallis non-parametric one-way analysis of variance across the two habitats (eelgrass versus unvegetated).

A. ALPHA DIVERSITY

A1. Shannon Index by Sample Type

**Kruskal-Wallis (all groups)**				
H	95.08390148			
p-value	1.77E-20			
**Kruskal-Wallis (pairwise)**				
**Group 1**	**Group 2**	**H**	**p-value**	**q-value**
biofilm (n=23)	marine sediment (n=24)	16.17798913	5.77E-05	5.77E-05
biofilm (n=23)	seawater (n=21)	29.07950311	6.95E-08	1.04E-07
biofilm (n=23)	whole gut (n=49)	46.31506849	1.01E-11	3.02E-11
marine sediment (n=24)	seawater (n=21)	32.86956522	9.86E-09	1.97E-08
marine sediment (n=24)	whole gut (n=49)	47.67567568	5.03E-12	3.02E-11
seawater (n=21)	whole gut (n=49)	26.2144705	3.06E-07	3.67E-07

A2. Shannon Index by Habitat

**Kruskal-Wallis (all groups)**		
H		0.162830806
p-value		0.686563523
q-value		0.686563523
**ANOVA**		
**Interaction term**	**F**	**p-value**
Benthic substrate*Sample type	1.62044	0.190648
Benthic substrate*Site	0.102657	0.958286

Supplemental Table B. Statistical tests and results for Beta Diversity using robust Aitchison Principal Component Analysis. Table B1 demonstrates Permanova test for RPCA distance across sample types with pairwise comparisons. Table B2 demonstrates an Adonis test to control for interaction between variables of benthic substrate (habitat), sample type, and geographic location (study site). Table B3 demonstrates Permanova test for RPCA distance between study sites (Fidalgo Bay, Case Inlet, Port Gamble, Skokomish) after filtering the biom table to only include oyster gut samples. Pairwise comparisons are also included.

B. BETA DIVERSITY

B1. RPCA differences between Sample Types

PERMANOVA (all groups)						
sample size (n)	117					
number of groups	4					
pseudo-F	123.43					
p-value	0.001					
number of permutations	999					
**PERMANOVA (pairwise)**						
**Group 1**	**Group 2**	**Sample size**	**Permutations**	**pseudo-F**	**p-value**	**q-value**
biofilm	marine sediment	47	999	84.01738318	0.001	0.001
biofilm	seawater	44	999	136.5532232	0.001	0.001
biofilm	whole gut	72	999	76.94670116	0.001	0.001
marine sediment	seawater	45	999	137.8757526	0.001	0.001
marine sediment	whole gut	73	999	123.43832	0.001	0.001
seawater	whole gut	70	999	218.8080857	0.001	0.001

B2. RPCA differences across Habitat + Sample Type + Site

Adonis ("permutational multivariate anova")						
Factor	Df	SumsOfSqs	MeanSqs	F.Model	R2	Pr(>F)
**benthic_substrate**	1	0.686711	0.686711	1.986832	0.002577	0.118
**sample_type**	3	203.873991	67.957997	196.619934	0.765052	0.001
**geo_loc_name**	3	24.249224	8.083075	23.386411	0.090997	0.001
**Residuals**	109	37.673808	0.345631		0.141374	
**Total**	116	266.483734			1	

Supplemental Table C. Statistical tests and results for comparison of Songbird differentials. Table C1 demonstrates a Kruskal Wallis non-parametric one-way analysis of variance for the ratio of *Vibrio*, *Synechococcus*, and Verrucomicrobiales to *Mycoplasma* and Desulfocapsaceae across study sites. This test was performed after running Songbird to produce the differentials of OTUs within only gut samples. The Dunn test is a post-hoc test to calculate pairwise distances of the ratio between each study site.

B3. RPCA differences between Sites (within Gut samples only)

**PERMANOVA (all groups)**						
sample size (n)	50					
number of groups	4					
pseudo-F	10.5873					
p-value	0.001					
number of permutations	999					
PERMANOVA (pairwise)						
**Group 1**	**Group 2**	**Sample size**	**Permutations**	**pseudo-F**	**p-value**	**q-value**
Fidalgo Bay	Case Inlet	32	999	4.313899854	0.024	0.024
Fidalgo Bay	Port Gamble	24	999	13.01511038	0.001	0.0015
Fidalgo Bay	Skokomish	28	999	15.99769319	0.001	0.0015
Case Inlet	Port Gamble	22	999	15.27254475	0.001	0.0015
Case Inlet	Skokomish	26	999	8.64054085	0.001	0.0015
Port Gamble	Skokomish	18	999	5.650489374	0.014	0.0168

C. SONGBIRD DIFFERENTIALS

Numerator: *g__Vibrio; g__Synechococcus* _CC9902; o__Verrucomicrobiales
Denominator: *g__Mycoplasma*;f__Desulfocapsaceae

C1. Comparison of Differential Ratios across Sites (within Gut samples only)

**Kruskal Wallis (all groups)**				
chi-squared	33.243			
df	3			
p-value	2.86E-07			
**Dunn test (pairwise)**				
**Group 1**	**Group 2**	**z-statistic**	**p-value**	**q-value**
FB	CI	2.896458	0.0019	0.0038
FB	PG	-4.880558	0	0
FB	SK	-4.843545	0	0
CI	PG	-2.313262	0.0104	0.0155
CI	SK	-2.204672	0.0137	0.0165
PG	SK	0.15843	0.4371	0.4371

Supplemental Table D. Statistical tests and results for comparison of temperature and dissolved oxygen across study sites for the 24-hour period immediately before sample collection. Table D1 demonstrates results from a permutational ANOVA for repeated measurements of dissolved oxygen across study sites and habitats. Table D2. demonstrates results from a permutational ANOVA for repeated measurements of temperature across study sites and habitats. A legend is included for Supplemental tables A-D.

D. Environmental Variables

D1. Dissolved Oxygen differences across Sites and Habitats

**PermANOVA** (Dissolved Oxygen ~ Site*Habitat + Error (repeated measures over time))									
	SSn	dfn	SSd	dfd	MSEn	MSEd	F	parametric P(>F)	permutation P(>F)
Site	7056	3	1299	143	2352.153	9.083	258.9586	0	0.0002
Habitat	8.354	1	1299	143	8.354	9.083	0.9197	0.3392	0.3266
Site*Habitat	22.19	3	1299	143	7.396	9.083	0.8143	0.488	0.4906

D2. Temperature differences across Sites and Habitats

**PermANOVA** (Temperature ~ Site*Habitat + Error (repeated measures over time))									
	SSn	dfn	SSd	dfd	MSEn	MSEd	F	parametric P(>F)	permutation P(>F)
Site	4228.6	3	489.9	143	1.41E+03	3.426	411.478	0	0.0002
Habitat	1.1509	1	489.9	143	1.15E+00	3.426	0.33596	0.5631	0.5626
Site*Habitat	0.2478	3	489.9	143	8.26E-02	3.426	0.02411	0.9949	0.9964

Legend:
q-value = adjusted p value using Benjamin Hochberg adjustment for multiple testing
Significant p values are highlighted in red. Significance level is < 0.05
* denotes an interaction term

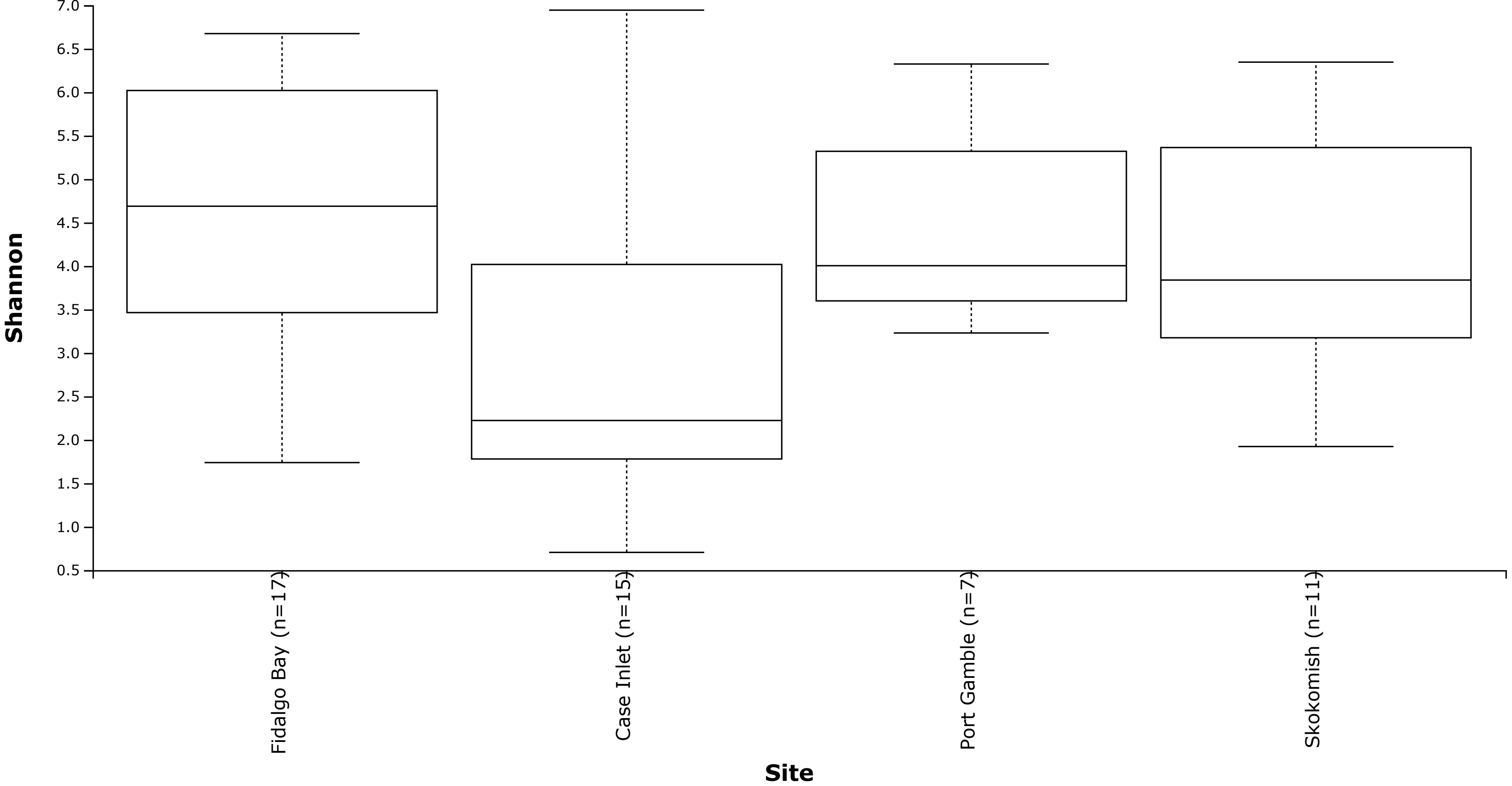

Kruskal-Wallis (all groups)				
H	5.009151485			
p-value	0.171128253			
**Kruskal-Wallis (pairwise)**				
		H	*p-value*	*q-value (p-adjusted)*
FB (n = 17)	CI (n = 15)	3.49411765	0.06158722	0.2268667
FB (n = 17)	PG (n = 7)	0.08168067	0.7750326	0.7750326
FB (n = 17)	SK(n = 11)	0.124469851	0.724236247	0.775032605
CI (n = 15)	PG (n = 7)	2.744099379	0.097614062	0.226866701
CI (n = 15)	SK(n = 11)	2.505723906	0.11343335	0.226866701
PG (n = 7)	SK(n = 11)	0.248120301	0.618401752	0.775032605

Supplemental Figure E. Boxplot demonstrating Shannon alpha diversity by study site after filtering the biom table to only include oyster gut samples. The statistical tests and results for the Shannon index comparison across study sites is included in the table. A Kruskal Wallis non-parametric analysis of variance was run on the oyster gut samples for differences in alpha diversity across study sites and a pairwise test was conducted to determine if oysters from any study sites significantly differed from one another.

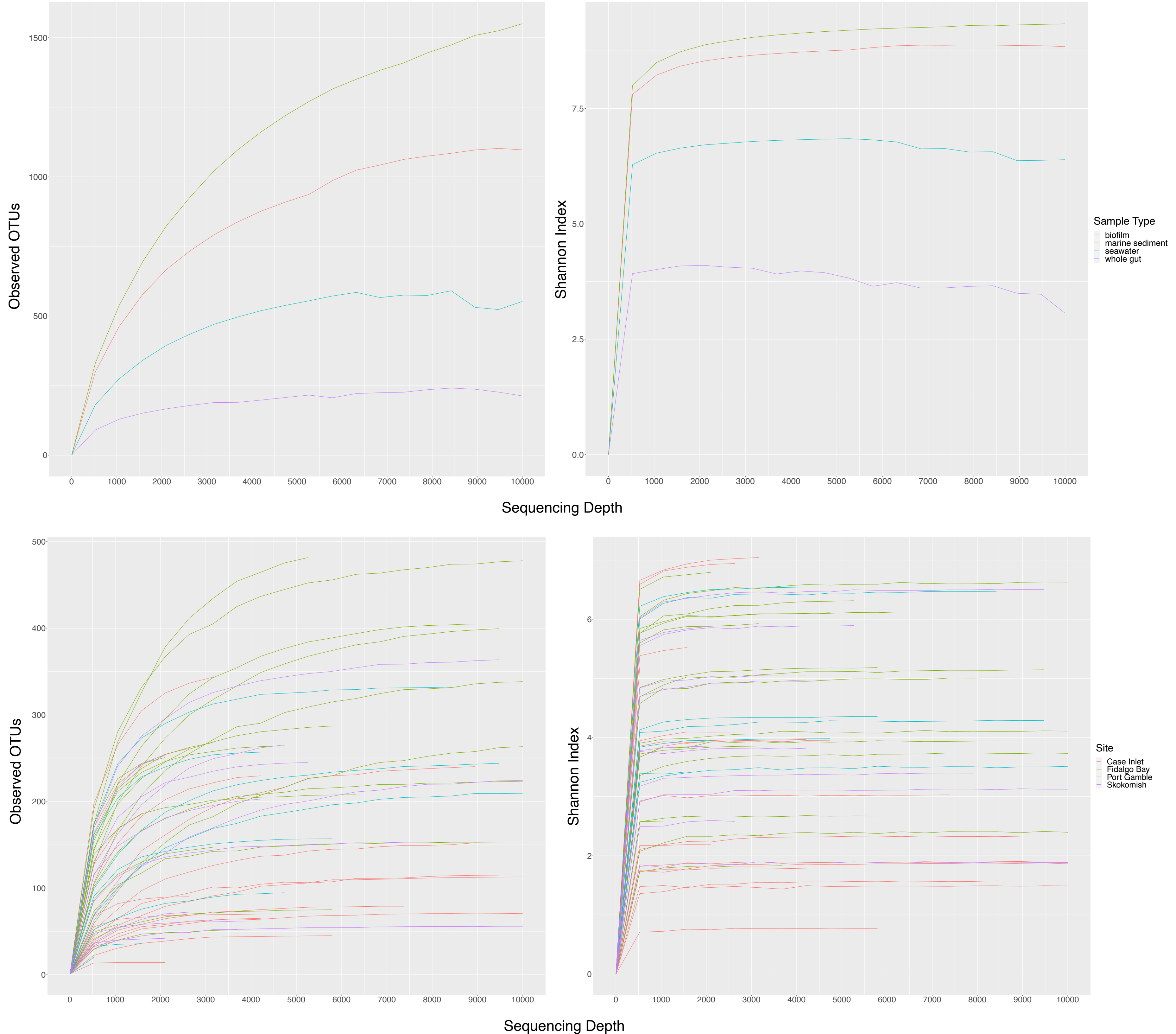

Observed OTUs
Shannon Index
Sequencing Depth
Sample Type
biofilm
marine sediment
seawater
whole gut
Site
Case Inlet
Fidalgo Bay
Port Gamble
Skokomish

Supplemental Figure F. Rarefaction curves for the number of OTUs observed and Shannon alpha diversity index with increasing sequencing depth. The top two plots contain lines which group (take the average) the number of OTUs or Shannon index values by sample type. The bottom two plots look only at oyster gut samples and show the number of OTUs or Shannon index values for each individual oyster and are colored by the site where the oyster was outplanted.
